# Choroidal shift in myopic eyes in the 10-year follow-up Beijing eye study

**DOI:** 10.1038/s41598-021-94226-0

**Published:** 2021-07-19

**Authors:** Jost B. Jonas, Yan Ni Yan, Qi Zhang, Rahul A. Jonas, Ya Xing Wang

**Affiliations:** 1 Beijing Institute of Ophthalmology, Beijing Tongren Hospital, Beijing Ophthalmology and Visual Sciences Key Laboratory, Capital Medical University, 1 Dongjiaomin Lane, Beijing, 100730 China; 2 Department of Ophthalmology, Medical Faculty Mannheim, Heidelberg University, Mannheim, Germany; 3 Institute of Molecular and Clinical Ophthalmology Basel, Basel, Switzerland; 4 Beijing Tongren Eye Center, Beijing Key Laboratory of Intraocular Tumor Diagnosis and Treatment, Beijing Ophthalmology and Visual Sciences, Key Lab, Beijing Tongren Hospital, Capital Medical University, Beijing, China; 5 Eye Center, The 2nd Affiliated Hospital, Medical College of Zhejiang University, Hangzhou, 310009 China; 6 Department of Ophthalmology, University Hospital of Cologne, Cologne, Germany

**Keywords:** Retina, Diseases, Eye diseases, Retinal diseases

## Abstract

The aim of the study was to assess longitudinal changes in the spatial relationship of the choroidal vasculature to retinal vasculature in myopic eyes. In the population-based longitudinal Beijing Eye Study in 2001/2011, we examined all highly myopic eyes with assessable fundus photographs and a randomized group of non-highly myopic. Using fundus photographs, we qualitatively assessed changes in the location of major choroidal vessels in relationship to retinal vessels. The study consisted of 85 highly myopic eyes (58 participants;age:64.8 ± 9.4 years) and 85 randomly selected non-highly myopic eyes. A choroidal shift in relationship to the retinal vessels was detected more often in the highly myopic group than the non-highly myopic group (47/85 (55%) vs 6/85 (7%); *P* < 0.001). In the highly myopic group, the choroidal vessel shift occurring on the disc-fovea line in 39 (44%) eyes, was similar to, or smaller than, the enlargement in gamma zone width in 26 (67%) eyes and in 11 (28%) eyes respectively. The choroidal vessel shift was larger (*P* = 0.002) in eyes without choroidal vessels in gamma zone than in eyes with large choroidal vessels in gamma zone. In 14 (17%) eyes, a localized centrifugal choroidal shift was observed in association with an increase in the stage of myopic maculopathy. The results suggest that highly myopic eyes show a change in the position of large choroidal vessels in relationship to retinal vessels, in association with development or enlargement of gamma zone and an increase in the stage of myopic maculopathy.

## Introduction

The axially elongating eye undergoes morphologic changes in its posterior hemisphere^1^. It includes a lengthening of the mid-peripheral region changing the spherical globe shape to an elongated form, and a posterior shift of Bruch´s membrane opening (BMO) in direction to the fovea^2,3^. The latter leads to an overhanging of Bruch´s membrane (BM) into the intrapapillary compartment at the nasal optic disc border, and to an absence of BM in the temporal parapapillary region, then called parapapillary gamma zone^3–5^. The posterior movement of the temporal border of the BMO is associated with a lengthening of the peripapillary choroidal border tissue which connects the end of BM with the peripapillary border tissue of the peripapillary scleral flange and indirectly with the lamina cribrosa^6^. Since the choriocapillaris is firmly connected through its basal membrane with BM, the posterior shift of the parapapillary BM may lead to a shift of the choriocapillaris parallel to the BM´s shift. Since the underlying layers of the medium-sized and large choroidal vessels are not firmly connected to the choriocapillaris, they may have some flexibility in their spatial relationship with BM, and they may not fully follow the backward movement of BM opening during the process of axial elongation. In previous studies, Moriyama and colleagues reported on differences in the choroidal architecture between highly myopic eyes with versus without posterior staphylomas. Using indocyanine green angiography, the authors found a displacement of the entry site of the posterior ciliary arteries into the choroid in about 75% of eyes with a posterior staphyloma versus 25% of eyes without staphyloma, and a lower number of large choroidal vessels in the posterior fundus of eyes with staphylomas^7^. Using wide-field indocyanine green angiography, Moriyama and associates also detected an association between a higher prevalence of posterior vortex veins and a higher prevalence of posterior staphylomas^8^. It was the purpose of our investigation to study in a longitudinal manner changes in the spatial relationship between the medium-sized/large choroidal vessel layer and BM, to assess differences in the axial elongation-associated backward movement of both tissue layers. We took the retinal vessels as surrogate of the position of BM. To avoid the risk of a referring bias, we chose a population-based recruitment of the study population.

## Methods

The Beijing Eye Study is a population-based longitudinal investigation carried out in the region of Greater Beijing^9^. The Medical Ethics Committee of Beijing Tongren Hospital approved the study protocol according to the declaration of Helsinki and all study participants gave their written informed consent. First conducted in 2001, the survey was repeated in 2011, inviting all participants from the survey of 2001.

All individuals participating in the study underwent a structured questionnaire and medical and ophthalmic examinations. The latter included measurements of visual acuity, slit lamp examination of the anterior and posterior ocular segment, and photography of the optic disc and macula (fundus camera Type CR6-45NM; Canon Inc., Tokyo, Japan). In 2011, optical coherent tomographic (OCT) images were taken of the optic nerve head and macula (Spectralis, Heidelberg Engineering Co, Heidelberg, Germany). As already described in detail previously, the protocol for imaging the optic disc and the macula included six radial scan lines with a scan length of 6 mm, centered on the optic disc and macula, respectively, and each comprising 100 A-scans^10^. The parapapillary region was examined with the intrinsic viewer (Heidelberg Eye Explorer software version 1.7.0.0; Heidelberg Engineering), which automatically synchronized the vertical lines of each B-scan and the infrared image taken by the OCT device. The parapapillary gamma zone was defined as the region between the end of BM and the border of the optic disc^5,10–12^. The optic disc border was defined as the end of the lamina cribrosa or as the inner side of the peripapillary border tissue of the peripapillary scleral flange. The OCT image of the macula served to determine the localization of the foveola, and the optic nerve head imaging served for defining the border of gamma zone. On the fundus photographs, we defined gamma zone as the parapapillary region, in which the sclera was clearly visible and covered by superficial retinal tissue (i.e. retinal vessels and retinal nerve fibers) while the choroid, except for few large choroidal vessels, was completely absent. The delineation of gamma zone on the fundus photographs was controlled by marking the border of gamma zone on the OCT images.

Aligning and flickering the photographs of the optic disc and macula, taken in 2001 and 2011, we assessed in a qualitative manner (yes /no) any changes in the position of the large choroidal vessels in relationship to the large retinal vessels, changes in the presence, size and location of parapapillary gamma zone, and changes in a diffuse chorioretinal atrophy as category 2 and patchy atrophies as category 3 of myopic macular degeneration^13^. In those eyes, in which a choroidal vessel shift occurred on the disc-fovea line, we additionally assessed in a semi-quantitative manner, whether the amount of choroidal shift was similar to, smaller than or larger than the enlargement in gamma zone width in the disc-fovea direction. The retinal images taken in 2011 were calibrated with respect to the baseline photos taken in 2001. For that purpose, fundus landmarks such as major vessel crossing were used to ensure that the photographs from 2001 and from 2011 were captured and compared from the same fundus region. These fundus landmarks did not include any aspect of parapapillary gamma zone or position of the large choroidal vessels. We did not measure the changes in the vessel positions or the changes in the size and shape of gamma zone in absolute measurement units such as millimeter since the alignment of the images taken in 2001 and in 2011 might have changed the image magnification of fundus elements by the optic media of the eye and by the fundus cameras.

Using a commercially available statistical software package (SPSS for Windows, version 25.0, IBM-SPSS, Chicago, IL, USA), we first calculated the mean and standard deviations of the demographic parameters. Using the chi-square test, we assessed associations between the occurrence of changes in the spatial relationship between the large choroidal versus retinal vessels, changes in gamma zone, and changes in the presence and size of a diffuse chorioretinal atrophy and macular BM defects^13,14^. All *P* values were two-sided and considered statistically significant when they were < 0.05.

## Results

The Beijing Eye Study conducted in 2011 included 2695 individuals who had also participated in the Beijing Eye Study performed in 2001. Out of 4439 individuals originally participating in the survey in 2001, there were 379 individuals who had died and 1365 individuals who had moved or did not want to be re-examined. The participants of the survey in 2011 had a mean age in 2011 of 64.7 ± 9.7 years (range: 50–93 years) and a mean refractive error of − 0.20 ± 2.13 diopters (range − 22.0 to + 7.5 diopters). Out of 204 highly myopic eyes with a myopic refractive error of ≥ − 6.0 diopters in the survey of 2001, 109 eyes were re-examined in the survey of 2011. For 24 of these eyes, the assessment of the choroid was not possible due to opacities of the fundus photographs, so that eventually 85 highly myopic eyes (58 participants; 35 (60%) women)) were included into the present study. The age of these participants (in 2011: 64.8 ± 9.4 years; median: 64 years; range 50–88 years) did not differ significantly (*P* > 0.10) from the age of the other participants of the survey (in 2011: 64.7 ± 19.7 years (median: 64 years; range 50–83 years)). The mean axial length was 27.6 ± 1.3 mm. Nine out of the 85 eyes were pseudophakic or aphakic. For the remaining eyes without cataract surgery, the mean refractive error was − 9.51 ± 3.11 diopters in 2011.

The present study additionally included a group of 85 randomly selected eyes out of the non-highly myopic group of the Beijing Eye Study. As described in detail previously, these eyes belonged to a group of healthy eyes which, stratified by refractive error subgroups of ± 1 diopter, had been randomly selected, which had been examined in 2001 and in 2011, and for which fundus photographs of sufficient quality for the detection of choroidal vessels were available^3^. The highly myopic group and the non-highly myopic group did not differ significantly in age (*P* = 0.23) and gender (*P* = 1.00) (Table 1).

**Table 1:** Demographics of the study population.

	Study group	Control group	
n (participants)	58	53	
n (eyes)	85	85	
Men/women	33/52	32/53	1.00
Age (years)	64.8 ± 9.4 (range: 50–88)	66.6 ± 9.7 (range: 50–88)	0.23
Axial length (mm)	27.6 ± 1.3 (range: 26.1–30.9)	23.6 ± 1.1 (range: 21.6–25.9)	< 0.001

**Figure 1:**
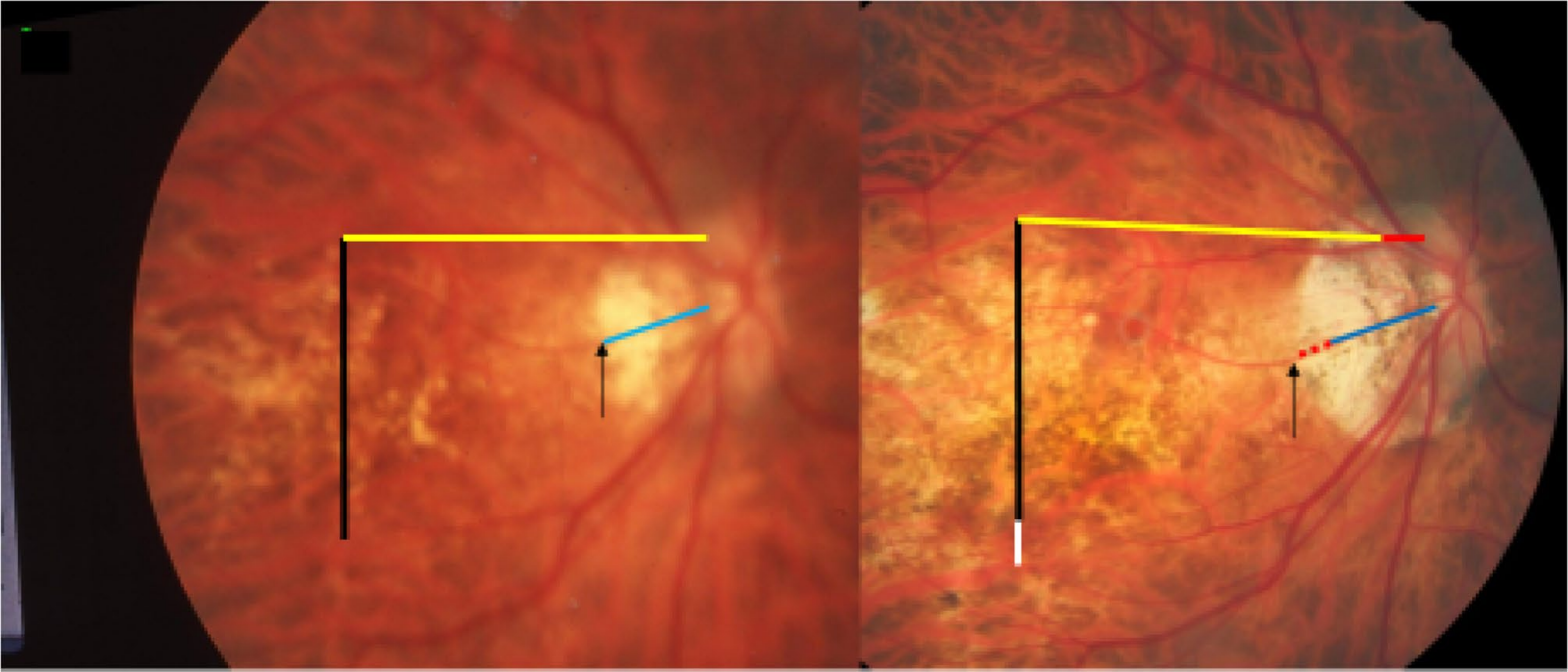
Fundus photographs of a highly myopic eye taken with an interval of 10 years (left image taken in 2001; right image taken in 2011). *Note*: Development and enlargement of a diffuse chorioretinal atrophy as category 2 of myopic macular degeneration. Elongation of the distance from a choroidal vessel marking to the optic disc border, compared between baseline (yellow bar) and follow-up (equally long yellow bar plus red bar); the elongation of the distance between the disc border and the choroidal marker is similar to the enlargement of parapapillary gamma zone without large choroidal vessels (black arrow) (2001: deep blue line; elongation in 2011: dotted red line); elongation of the distance between two choroidal vascular markings, compared between baseline (vertical black bar) and follow-up (equally long vertical black bar plus white bar);

**Figure 2:**
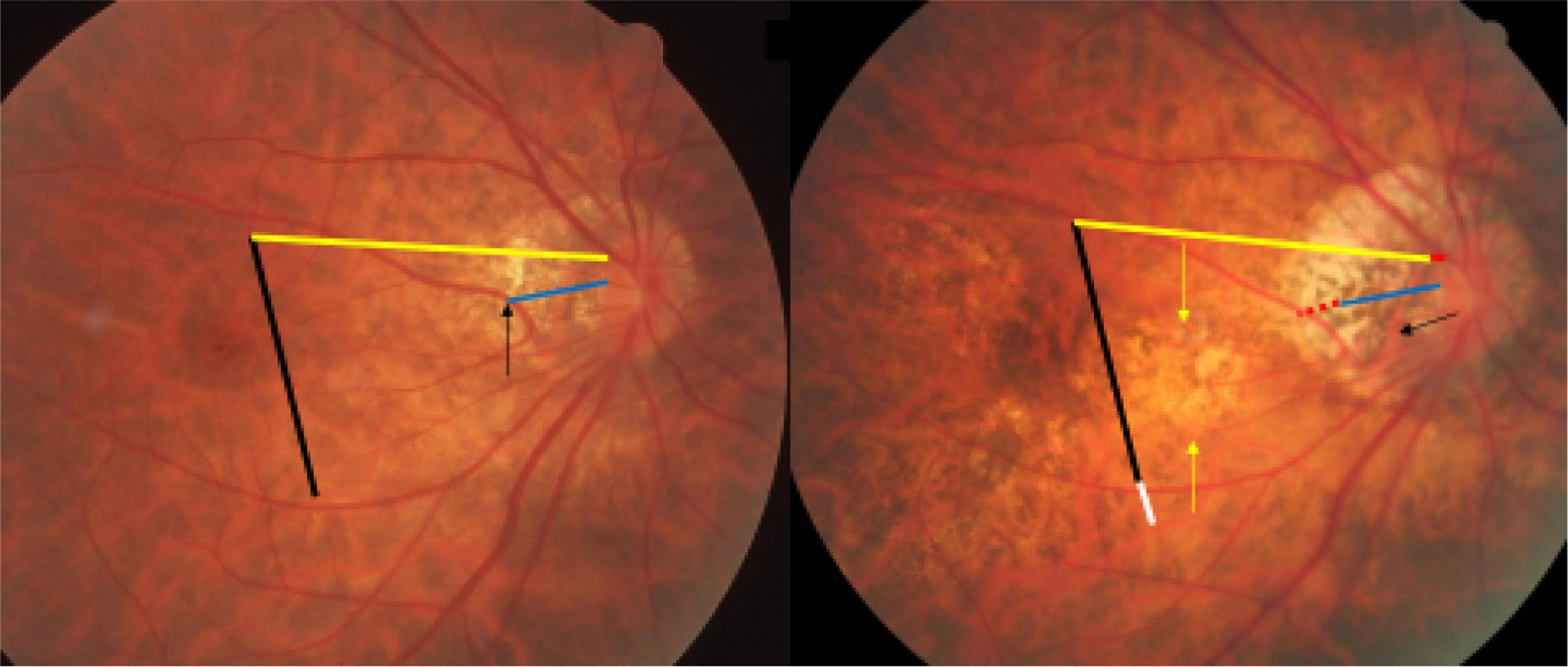
Fundus photographs of a highly myopic eye taken with an interval of 10 years (left image taken in 2001; right image taken in 2011). *Note*: Development and enlargement of a diffuse chorioretinal atrophy as category 2 of myopic macular degeneration (yellow arrows). Elongation of the distance from a choroidal vessel marking to the optic disc border, compared between baseline (yellow bar) and follow-up (equally long yellow bar plus red bar); the elongation of the distance between the disc border and the choroidal marker (red bar) is smaller than the enlargement of parapapillary gamma zone with large choroidal vessel tracings (black arrow) (2001: deep blue line; elongation in 2011: dotted red line); elongation of the distance between two choroidal vascular markings, compared between baseline (vertical black bar) and follow-up (equally long vertical black bar plus white bar), perpendicular to the longest axis of the diffuse chorioretinal atrophy region.

A shift of large choroidal vessels in relationship to the retinal vessels was observed significantly (*P* < 0.001) more often in the highly myopic group (in 47/85 (55%) eyes) than in the non-highly myopic group (in 6/85 (7%) eyes). In the highly myopic group, the choroidal vessel shift occurred on the disc-fovea line in 39 (44%) of the 85 highly myopic eyes. In 26 (67%) of the 39 eyes, the choroidal shift was similar to the enlargement in the width of gamma zone; in 11 (28%) eyes the choroidal shift was smaller than the gamma zone enlargement; and in 2 (5%) eyes, the choroidal shift occurred without a change in gamma zone size (Figs. 1, 2).

In 9 (82%) of the 11 highly myopic eyes with a choroidal shift smaller than the gamma zone enlargement, few large choroidal vessels were present in gamma zone (Fig. 2). Within the group of the 39 highly myopic eyes with a choroidal shift on the disc-fovea line, the choroidal shift in eyes without choroidal vessels in gamma zone as compared with eyes with large choroidal vessels in gamma zone was significantly more often similar to the gamma zone enlargement (19/22 (86%; 95% CI 71, 100); *P* = 0.002) versus 5/15 (33%; 95% CI 6.3, 60.4)), while it was in a significantly lower frequency smaller than the gamma zone enlargement (2/22 (9%; 95% CI 0, 22); *P* = 0.002) versus 9/15 (60%; 95% CI 32, 88)) (Figs. 1, 2). Within the group of the 39 highly myopic eyes with a choroidal shift in the disc-fovea line, 9 (23%) eyes showed a localized increase in the choroidal inter-vessel distance, 10 (26%) eyes showed a localized decrease in the choroidal inter-vessel distance, and one eye showed a mix of localized increase and localized decrease.

**Figure 3:**
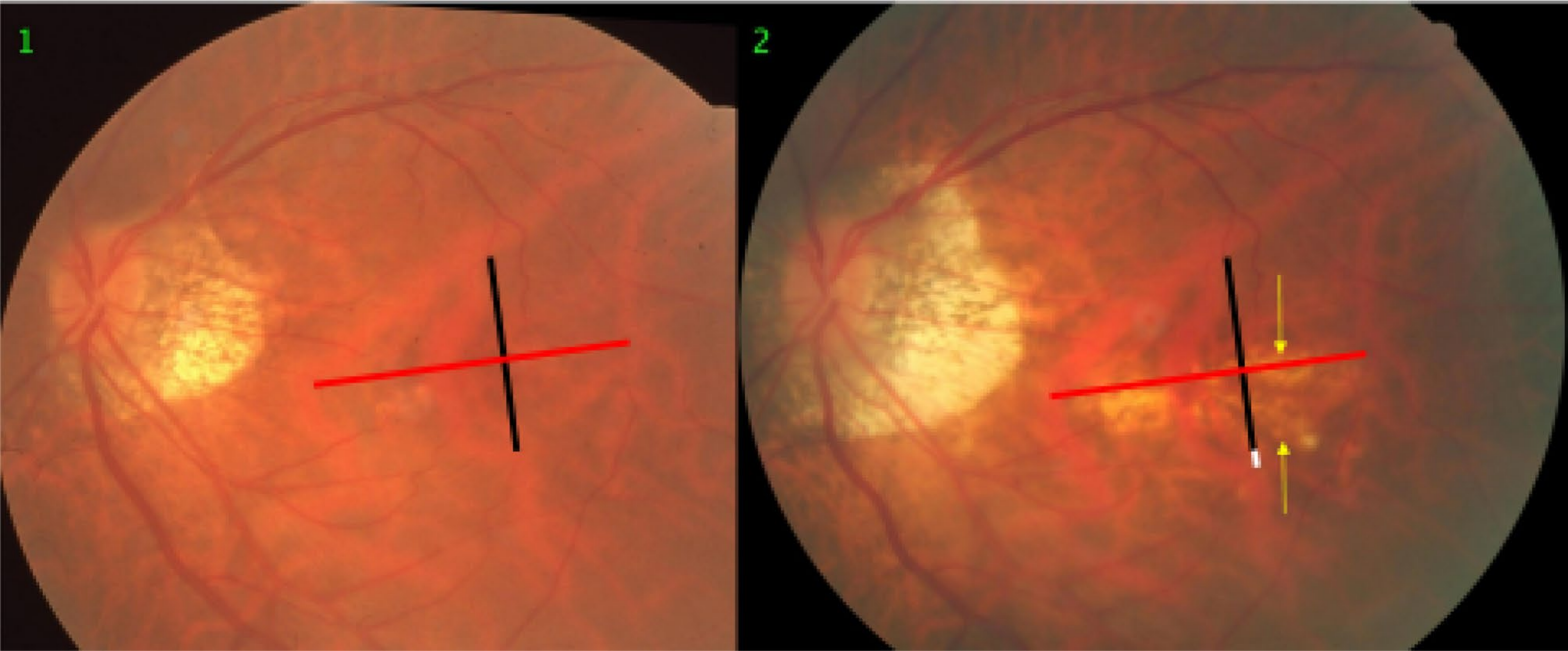
Fundus photographs of a highly myopic eye taken with an interval of 10 years (left image taken in 2001; right image taken in 2011). *Note*: Development and enlargement of a diffuse chorioretinal atrophy (yellow arrows) as category 2 of myopic macular degeneration. Elongation of the distance between two vertical choroidal vascular markings, compared between baseline (vertical black bar) and follow-up (equally long vertical black bar plus white bar); no change in the distance between two horizontal markings (equally long red bars). The choroidal vessel shift occurred perpendicular to the longest diameter of the area with diffuse chorioretinal atrophy.

**Figure 4:**
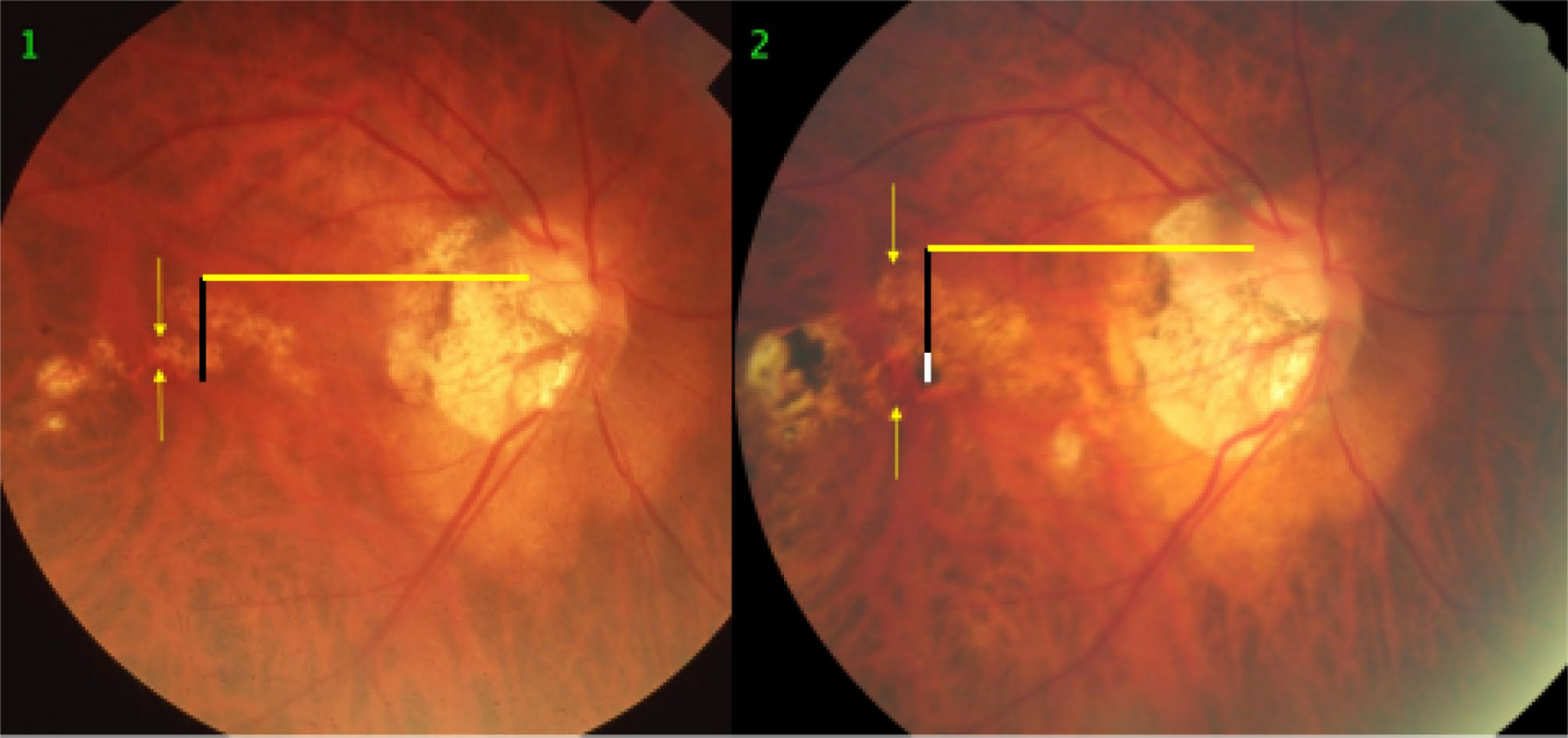
Fundus photographs of a highly myopic eye taken with an interval of 10 years (left image taken in 2001; right image taken in 2011). *Note*: enlargement of a diffuse chorioretinal atrophy /lacquer crack (yellow arrows) as category 2 of myopic macular degeneration. Elongation of the distance between two choroidal vascular markings, compared between baseline (vertical black bar) and follow up (equally long vertical black bar plus white bar), suggesting a spreading of the choroidal vessels. Distance between the choroidal marking and the disc border (equally long yellow bars) constant during follow-up. The choroidal vessel shift occurred perpendicular to the longest diameter of the area with diffuse chorioretinal atrophy/lacquer crack.

**Figure 5:**
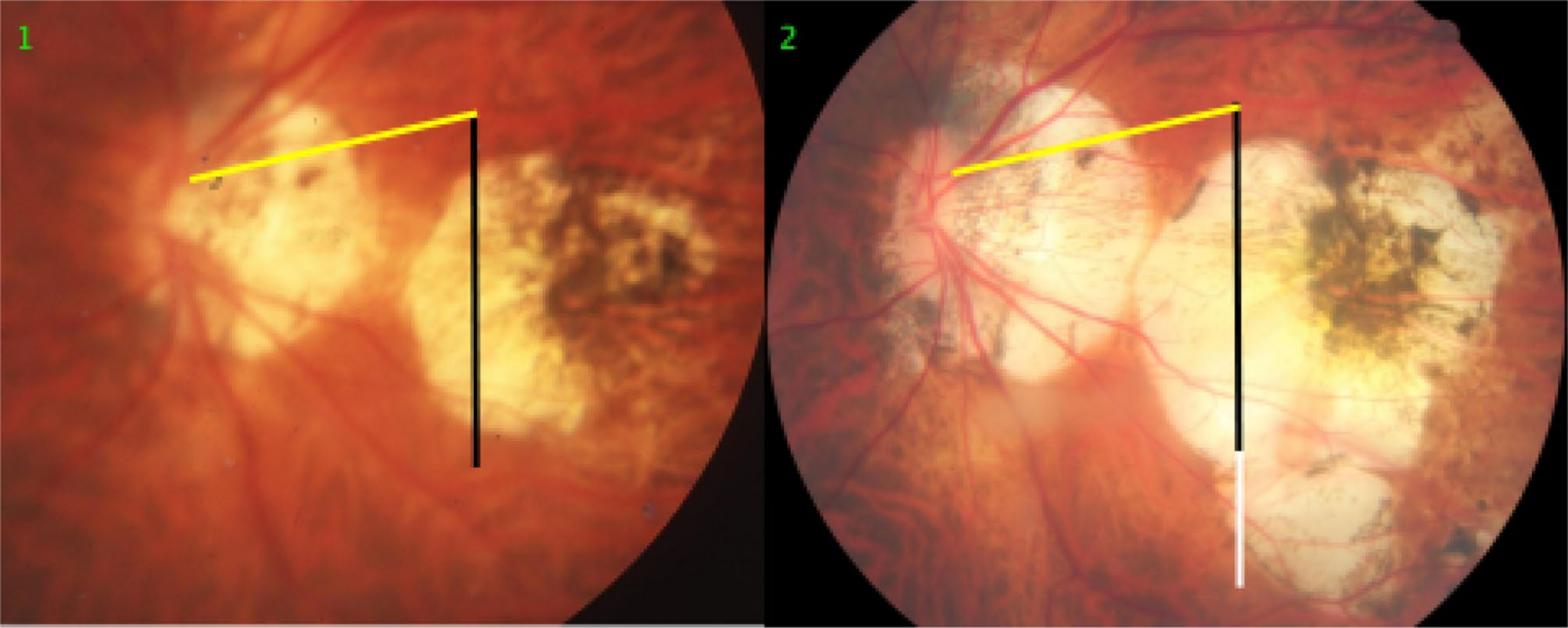
Fundus photographs of a highly myopic eye taken with an interval of 10 years (left image taken in 2001; right image taken in 2011). *Note*: enlargement of the foveal Bruch´s membrane defect (category 4 of myopic macular degeneration) without underlying large choroidal vessels). Elongation of the distance between two vertical choroidal vascular markings, compared between baseline (vertical black bar) and follow up (equally long vertical black bar plus white bar), suggesting a spreading of the choroidal vessels and corresponding in size to the vertical enlargement of the Bruch’s membrane defect. Distance between the choroidal marking and the disc border (equally long yellow bars) constant during follow-up.

**Figure 6:**
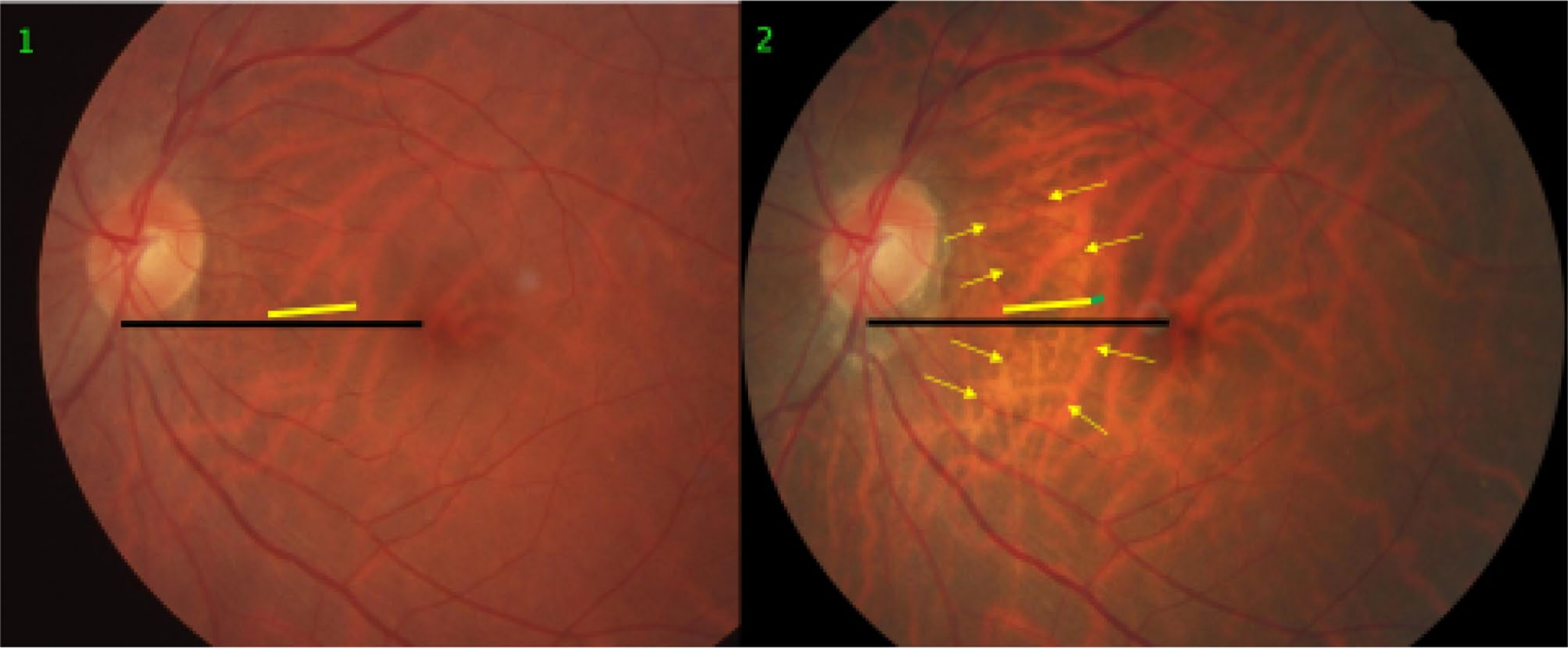
Fundus photographs of a highly myopic eye taken with an interval of 10 years (left image taken in 2001; right image taken in 2011). *Note*: development of a vertical diffuse chorioretinal atrophy (multiple yellow arrows), parallel to an elongation of the distance between two horizontal choroidal vascular markings, compared between baseline (yellow bar) and follow up (equally long yellow bar plus green bar), suggesting a spreading of the choroidal vessels. Distance between the fovea and the disc border (equally long black bars) constant during follow-up. The choroidal vessel shift occurred perpendicular to the longest diameter of the area with diffuse chorioretinal atrophy.

**Figure 7:**
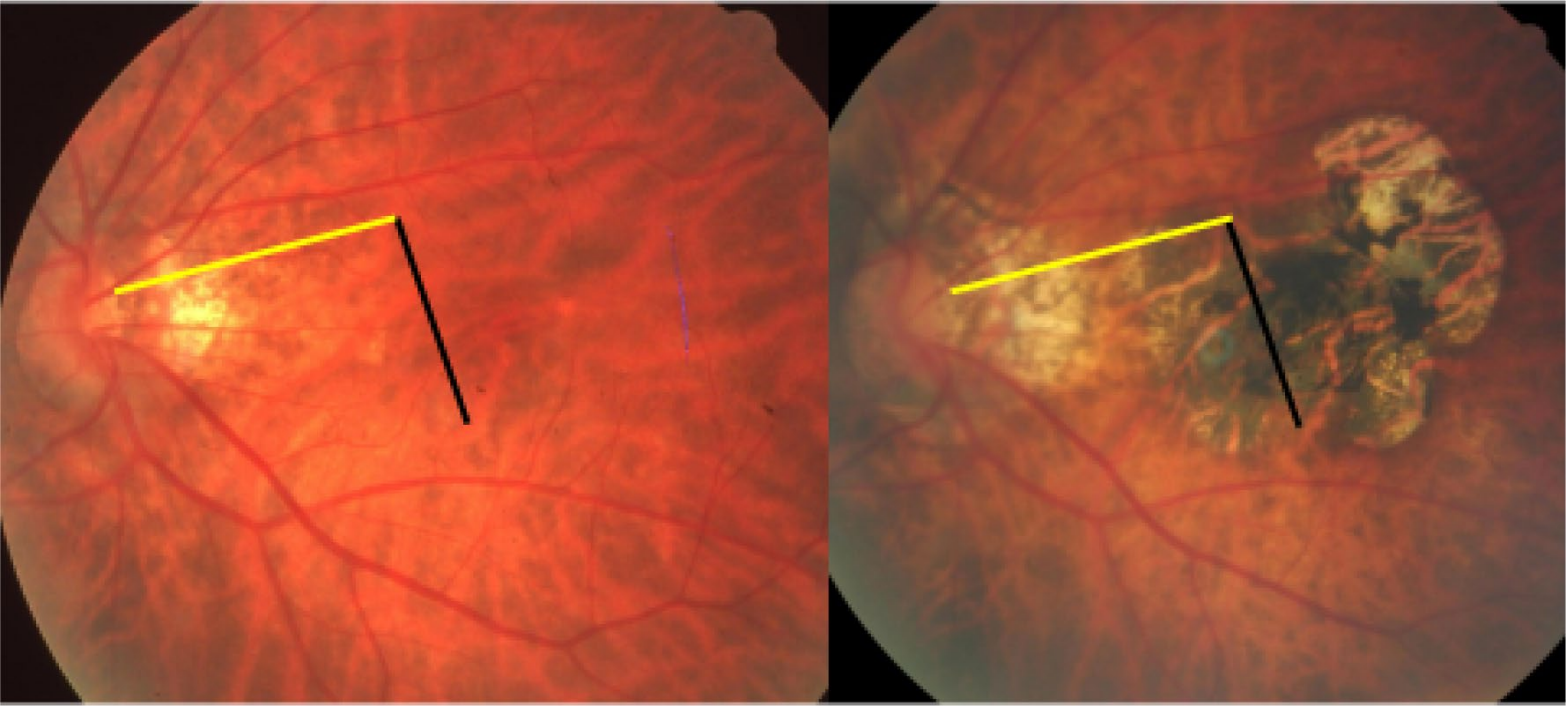
Fundus photographs of a highly myopic eye taken with an interval of 10 years. *Note*: development of a foveal Bruch’s membrane defect (as category 4 of myopic macular degeneration) with an apparent intact large choroidal vessel layer. No change in the distance between two choroidal vascular markings, compared between baseline (black bar) and follow up (equally long vertical black bar), and no change in the distance between the choroidal marking and the disc border (equally long yellow bars).

In 14 (17%) eyes, a choroidal shift was observed in association with the development or enlargement of a diffuse chorioretinal atrophy or a macular BM defect (Figs. 1, 2, 3, 4, 5, 6). In 10 (71%) of these 14 eyes, there was an additional choroidal shift in the disc-fovea line. The BM defect-associated choroidal shift showed a centrifugal movement of the choroidal vessels away from the center of the BM defect in eyes with a mostly circular BM defect. In the case of an elongated BM defect, the shift of the choroidal vessels occurred in a direction perpendicular to the longest diameter of the lesion. In eyes with a macular BM defect with visible choroidal vessels at its ground, the inter-vessel distance in the choroidal large vessel layer did not markedly change (Fig. 7).

In the non-highly myopic group, the choroidal vessel shift occurred on the disc-fovea line in 6 (7%) of the 85 non-highly myopic eyes. In 5 (83%) of the 6 eyes, the choroidal shift was similar to the enlargement in the width of gamma zone; in one (17%) eye, the choroidal shift was smaller than the gamma zone enlargement. Since BM defects did not occur in the non-highly myopic eyes, there were no choroidal changes in association with BM defects in the non-highly myopic group.

## Discussion

In this population-based longitudinal study, a shift of large choroidal vessels in relationship to retinal vessels was observed in 47 (55%) out of 85 highly myopic eyes. The shift occurred most often in the disc-fovea line, and corresponded to the gamma zone enlargement in eyes with a parapapillary gamma zone without choroidal vessels. In eyes in which gamma zone showed some large choroidal vessels, the choroidal shift was smaller than the enlargement of gamma zone. In the region of a developing or enlarging diffuse chorioretinal atrophy or a macular BM defect, the choroidal inter-vessel distance increased, suggesting a choroidal vessel shift or spreading in a direction perpendicular to the longest diameter of the lesion. In eyes with a macular BM defect with choroidal vessels, the inter-vessel distance in the choroidal large vessel layer did not markedly change.

The observation made in this study cannot directly be compared with findings of other investigations since a change in the arrangement of the large choroidal vessels in highly myopic eyes has not yet been intensively examined yet. Many studies have reported on a thinning of the choroid overall, and some studies have found that the axial elongation-associated choroidal thinning mainly affected the medium-sized vessel layer and the large-vessel layer, while the thickness of the small-vessel layer and the thickness of the choriocapillaris did not show major changes or no change at all, respectively^15–17^.

The findings made in our study can be divided into changes in the position of the large choroidal vessels occurring in association with an enlargement of parapapillary gamma zone, and into those found in association with a developing or enlarging diffuse chorioretinal atrophy or macular BM defect. It has been discussed that the development of gamma zone in moderately myopic eyes is due to a shift of the BM opening of the optic nerve head into the temporal direction, leading to an overhanging of BM into the intrapapillary compartment at the nasal disc border, and to a compensatory lack of BM (i.e., gamma zone) in the temporal parapapillary region^3,4,18^. Since the large choroidal vessels are not connected with the underlying sclera (except for the few vortex veins in the equatorial region, and except for the short ciliary arteries in the peripapillary region), one may assume that the large choroidal vessels can shift on the sclera in a relatively unrestricted manner. The large and medium-sized choroidal vessels are connected with the choriocapillaris, which is firmly attached to BM through its basal membrane. One may postulate, that any shift in BM will lead to a similar shift of the choriocapillaris, and more or less to a similar shift of the large choroidal vessels. Since the retinal vessels are connected with the optic nerve head and get even slightly stretched by the gamma zone-associated elongation of the disc-fovea distance, their gamma zone enlargement-associated shift is limited^11^. It can lead to a change in the relative position of the retinal vessel-based landmarks compared with the position of large choroidal vessel-associated landmarks, as observed in the present study (Figs. 1, 2, 3, 4, 5, 6). The large choroidal vessels are fed by, and are connected with, the short posterior ciliary arteries which penetrate the sclera in the peripapillary region. One observation made in the present study was that the choroidal vessel shift was less marked in eyes which showed large choroidal vessels in their gamma zone. One may assume that in these eyes, the peripapillary ciliary artery system prevented a full shift of the large choroidal vessel layer. It suggests that in some highly myopic eyes with enlarging gamma zone a strain within the large choroidal vessel layer may develop, due to a BM-associated shifting of the choriocapillaris while the peripapillary ciliary arteries in the peripapillary region prevented a full shift of the large choroidal vessels. It may also suggest that within the retinal layers a strain develops between the inner retinal layers with the retinal vessels and the retinal nerve fibers both connected with the optic disc, and the deep retinal layers with the retinal photoreceptors firmly connected to the retinal pigment epithelium and indirectly to BM.

Independently of the gamma zone enlargement, a shifting of the large choroidal vessels also occurred in regions with a developing or enlarging diffuse choroidal atrophy and macular BM defects (Figs. 1, 2, 3, 4, 5, 6). In the case of an developing and enlarging diffuse chorioretinal atrophy, it means an increasing shearing strain between the large choroidal vessels and the choriocapillaris, since the position of BM and the adherent choriocapillaris is unchanged in the region of a diffuse chorioretinal atrophy. In eyes with an enlarging macular BM defect, the shearing strain between the choriocapillaris and the large choroidal vessels present in the region of a diffuse chorioretinal atrophy may be absent since the area of a BM defect does not contain a choriocapillaris. In the region of a BM defect however, the inner retinal layers are intact while the outer retinal layers, i.e., the photoreceptor layer with the adherent RPE and BM shifted and are not present in the BM defect. It may lead to an intraretinal strain between the outer retinal layers at the border of the BM defect and the inner retinal layers in the center of the BM defect. One may discuss whether such a strain may be one out of several factors leading to a myopic maculoschisis and to a loss of retinal cells. Interestingly, some BM defects preserved the pattern of the large choroidal vessel layer, in their full area, or segmentally in some parts of their area (Fig. 7). The shifting of the large choroidal vessels may thus not be a necessary condition for the development of a macular BM defect, and may just be an epiphenomenon.

Limitations of our study should be considered, when its findings are discussed. First, only 85 out of 204 highly myopic eyes which were examined in the survey in 2001 were included into the present study. This figure of a re-participation and re-assessment of 42% in a 10-year follow-up study is relatively low, even for a 10-year follow-up investigation. The data on the incidence of a choroidal vessel shift are therefore less solid than is the observation of the phenomenon as such. Second, we used choroidal landmarks such as crossings of large choroidal vessels or other particularities of the large choroidal vessel architecture to assess changes in the position of the choroidal vessels during follow-up. We chose those landmarks which were the best to be located at baseline and to be re-located at the follow-up examination. These landmarks were thus not representative of the whole choroidal vascular architecture, so that the results of our study may serve more as prove of principle than as a representative quantitative analysis of all positional choroidal vascular changes during the follow-up. In particular, one may take into account that the positional change of the choroid may differ between various regions, in particular in areas with a scleral staphyloma, and one may consider that large choroidal vessels, and thus their change, can be difficult to be detected in non-highly myopic eyes. Third, we did not perform quantitative measurements of the observed differences between the baseline images and the follow-up images. Although it would have given more detailed data, the main purpose of the study was to assess, as proof of principle, whether there were changes at all in the spatial relationship between the choroidal vessels and retinal vessels. Future studies may quantify such changes and assess linear relationships between the quantitative parameters. Fourth, we did not take indocyanine green angiographic images which would have facilitated the detection of the large choroidal vessels. Due to practical reasons, however, it is not feasible to take angiograms from participants of a population-based study. Fifth, the age of the study participants was 50 + years, so that the high myopia assessed in our investigation may be different from the type of “school children myopia” common in today´s young generation. Future studies may address whether similar observation on a shift of the large choroidal vessels with further axial elongation can be made in young individuals with progressive myopia. Such studies may also explore whether observations obtained in our Chinese population can be transferred on population of different ethnicities.

In conclusion, highly myopic eyes show a change in the position of large choroidal vessels in relationship to retinal vessels, in association with development or enlargement of gamma zone, diffuse chorioretinal atrophy or macular Bruch´s membrane defects.
